# Elucidating Plant-Microbe-Environment Interactions Through Omics-Enabled Metabolic Modelling Using Synthetic Communities

**DOI:** 10.3389/fpls.2022.910377

**Published:** 2022-06-20

**Authors:** Ashley E. Beck, Manuel Kleiner, Anna-Katharina Garrell

**Affiliations:** ^1^Department of Biological and Environmental Sciences, Carroll College, Helena, MT, United States; ^2^Department of Plant and Microbial Biology, North Carolina State University, Raleigh, NC, United States

**Keywords:** synthetic communities, plant microbiome, plant microbial interactions, metabolic modelling, flux balance analysis, elementary flux mode analysis

## Abstract

With a growing world population and increasing frequency of climate disturbance events, we are in dire need of methods to improve plant productivity, resilience, and resistance to both abiotic and biotic stressors, both for agriculture and conservation efforts. Microorganisms play an essential role in supporting plant growth, environmental response, and susceptibility to disease. However, understanding the specific mechanisms by which microbes interact with each other and with plants to influence plant phenotypes is a major challenge due to the complexity of natural communities, simultaneous competition and cooperation effects, signalling interactions, and environmental impacts. Synthetic communities are a major asset in reducing the complexity of these systems by simplifying to dominant components and isolating specific variables for controlled experiments, yet there still remains a large gap in our understanding of plant microbiome interactions. This perspectives article presents a brief review discussing ways in which metabolic modelling can be used in combination with synthetic communities to continue progress toward understanding the complexity of plant-microbe-environment interactions. We highlight the utility of metabolic models as applied to a community setting, identify different applications for both flux balance and elementary flux mode simulation approaches, emphasize the importance of ecological theory in guiding data interpretation, and provide ideas for how the integration of metabolic modelling techniques with big data may bridge the gap between simplified synthetic communities and the complexity of natural plant-microbe systems.

## Introduction

Plants have evolved intricate signalling networks sensing and responding to environmental stimuli. In recent years, the complex network of interactions existing between plants, microorganisms, and their environment has been recognized as an important factor impacting plant health and productivity (Timm et al., 2018; Kumar and Verma, 2019; Harman et al., 2021). Plants rely on microbes for biologically available forms of essential nutrients (Richardson et al., 2009); beneficial microbes also aid in protecting against pathogens and pests and are involved in plant response to environmental conditions such as heat and drought (Bhattacharyya et al., 2016; Liu et al., 2020).

The numerous multi-directional interactions that occur among plants, colonizing microorganisms, and environmental variables create difficulty in determining cause-and-effect relationships. For example, soil contains thousands of different microbial taxa (Lennon et al., 2012), a subset of which are recruited for colonization by plant secretion of root exudates (e.g., Lebeis et al., 2015); in addition to chemical signalling factors from roots, environmental factors such as soil chemical properties, pH, moisture content, and temperature also affect microbial colonization (Andrew et al., 2012; Islam et al., 2020b), influencing both microbe-microbe and plant-microbe interactions. Synthetic communities provide a reductionist approach to help constrain biological factors, examining a limited number of microbial species with key roles in the community to better understand driving forces and their overall effect on the productivity and resilience of plant-microbe systems (Vorholt et al., 2017). In the last decade, synthetic communities have become widely applied in a variety of contexts including agriculture (De Roy et al., 2014; De Souza et al., 2020; Sgobba and Wendisch, 2020), recognizing their value in experimental hypothesis testing.

Even with the tools provided by synthetic communities, an accurate understanding of multi-species, cross-domain interactions is still complicated by the complexity of each individual member’s metabolic network. Metabolic modelling provides mathematical predictions of metabolic routes used by an organism under different biological and environmental constraints and can thus help quantify the function of individual members and identify potential roles in community interaction on a metabolic level (Bosi et al., 2017). This technique complements experimental findings with a computational aspect that can aid in elucidating interactions that may elude solely experimental approaches. This perspectives article provides insight into how metabolic modelling can be integrated with experiments, using ecological theories to mine the complexities of plant-microbe-environment interactions and illuminate underpinning rules that lead to increased productivity and robustness in these systems.

## Overview of Synthetic Communities in Plant Systems

Synthetic communities have been developed for a variety of plants to provide more tractable systems for controlled lab-scale experiments. Microbes have been isolated and cultured from several different plant species of interest, including the model plants *Arabidopsis thaliana* and *Lotus japonicus*, and agriculturally important species such as rice, maize, tomato, potato, clover, and sugarcane (Liu et al., 2019; Wippel et al., 2021), identifying key functional microbial taxa that may influence plant-microbe interactions. Synthetic microbial communities have been developed for both rhizosphere and phyllosphere communities in several species: most notably *Arabidopsis*, as well as maize, sorghum, and duckweed, among others, with varying levels of phylogenetic representation (Table 1). Varied approaches have been taken for designing synthetic communities with the ultimate goal of developing a community representative of the function and interactions of a natural, complex microbiome (e.g., Timm et al., 2016; Wang et al., 2021). Co-occurrence network analyses are commonly used to identify core functional microbiomes – a reduced subset of microbial species that captures the main metabolic processes performed by a community (Toju et al., 2020), but other methods recognize the inherent complexity of non-equilibrium microbe-environment interactions and incorporate time-series data with empirical methods (Deyle et al., 2016; Suzuki et al., 2017). Metabolic modelling provides a theoretical approach to complement the experimental process of first selecting and building a synthetic community, as well as predicting physiological properties of the community.

**Table 1:** Highlighted synthetic microbial communities and community metabolic models.

Development of select synthetic microbial communities			
Species	Number of members	Bacterial phyla represented	Reference
*Arabidopsis* phyllosphere	62	9	Carlström et al., 2019
*Arabidopsis* rhizosphere	185	4	Finkel et al., 2019
Maize	7	3	Niu et al., 2017
Sorghum	36	4	Chai et al., 2021
Duckweed	6	2	Ishizawa et al., 2020
**Select microbial community metabolic models**			
**Environment**	**Findings**		**Reference**
Sulfate-reducing bacterial community	Illuminated the important role of hydrogen in syntrophic exchange between sulfate reducer and methanogen		Stolyar et al., 2007
International Space Station microbiome	Investigated potential interactions of dominant *Klebsiella pneumoniae* – mutualistic interactions with other bacteria and negative interactions with fungi		Kumar et al., 2021
Washington lake environmental communities	Demonstrated differential responses by two major methanotrophic species to environmental factors (carbon, oxygen, nitrogen levels)		Islam et al., 2020a
Human gut microbiome	Demonstrated the spatial organization and response of microbial community members based on oxygen availability in wound colonization		Chan et al., 2019
Synthetic microbiome colonizing the mouse gut	Utilized metabolomics data in combination with models to determine type and directionality of interactions, and demonstrated change in community composition as a function of nutritional environment		Weiss et al., 2021

Select synthetic microbial communities have been developed for a number of plant species, encompassing an array of different bacterial phyla. Select recent studies using microbial community models have uncovered new functionalities and interactions previously unknown by experimental means.

## What Is Metabolic Modelling?

A metabolic model is a mathematical representation of the stoichiometries of metabolic reactions occurring within an organism, ranging in complexity from prokaryotes to eukaryotes. Models are generated from genomic data using databases such as KEGG, MetaCyc, JGI IMG/M, RAST, and BRENDA to identify genes and associated metabolic reactions, collect metabolite stoichiometries for each reaction, and compile the information into a metabolite-reaction stoichiometry matrix *via* software tools such as KBase, Python, or CellNetAnalyzer (von Kamp et al., 2017; Arkin et al., 2018; Medlock et al., 2020). The process of building a metabolic model is a type of experiment in itself, as model accuracy is influenced by genome completeness, quality of genomic data and annotations (which can result in missing genes, reactions, or pathways), and availability of experimental data (such as biomass composition and maintenance energy requirements). The outcome of metabolic model construction is a hypothesis that can be used to predict physiological response under different environmental circumstances.

Simulation methods vary in their mathematical approach to solving the system of linear equations produced by the metabolite-reaction stoichiometric matrix, but all assume a steady state environment (*Sv* = 0, where *S* is the stoichiometric matrix and *v* is the flux vector). This system of equations is typically underdetermined due to a greater number of participating metabolites than number of reactions (Klamt et al., 2002); therefore, infinitely many flux solutions may satisfy the system. Elementary flux mode analysis and flux balance analysis are two of the most commonly employed simulation approaches and differ in computational intensity and scope of the solutions obtained, providing different advantages for plant microbiome applications.

Elementary flux mode analysis is a computationally intensive approach that enumerates all possible minimal pathways that balance the stoichiometric matrix (i.e., if a single reaction were removed from a minimal flux vector, the route through the network would be incomplete). This approach has been likened to finding the most efficient path when navigating a subway network (Zanghellini et al., 2013). From the entire set of elementary flux modes, any possible metabolism of the organism can be described by a linear combination of the elementary flux modes, serving as a comprehensive and unbiased examination of the organism’s physiological potential. The number of modes calculated for a given model depends on the model structure (reaction connectivity and reversibility, exchange reactions, etc.); but computational expense typically limits applications to small networks, though recent algorithm developments have enabled computation of 12 billion elementary flux modes (Buchner and Zanghellini, 2021). For complex communities, feasibility may be improved by performing elementary flux mode analysis for each species model separately, individually analyzing results for biologically and ecologically relevant pathways (e.g., Beck et al., 2017). The curated results can then be transferred into a less expensive optimization method (like flux balance analysis described below), similar to the hybrid analysis method demonstrated in Hunt et al. (2018), to enhance simulation tractability for plant-microbe systems.

Alternatively, flux balance analysis is a less computationally intensive approach using a linear optimization strategy to solve the stoichiometric matrix. By applying constraints to cellular uptake and secretion reactions along with an objective criterion (typically maximization of biomass production), the metabolic network is optimized under a restricted space. While this method is more easily applied to large networks, it presents bias toward the researcher-determined objective criterion, and it often does not provide a unique solution as more than one flux vector may achieve the same optimized objective value due to network redundancy. Flux balance analysis provides great utility in specifying uptake and secretion rates of nutrients and metabolic byproducts and can be used to test hypotheses of metabolite exchange *in silico*, which is key to elucidating plant-microbe and microbe-microbe interactions. Several algorithmic variations have extended this approach to examine more complex cellular environments, allowing consideration of other constraints on metabolism that are also useful to plant systems, such as cellular volume and biosynthetic costs of enzyme assembly (Mori et al., 2016), intracellular heterogeneity (Damiani et al., 2017), balance of resource usage (Goelzer and Fromion, 2011), and dynamic growth and integration with consumer-resource models (Mahadevan et al., 2002; Gowda et al., 2022).

### How Can Metabolic Modelling Be Applied to Understand Multi-Organism Interactions?

While metabolic modelling was initially employed for singular species, increased availability of genomic data and development of more efficient computational algorithms has enabled application to community models. Stolyar et al. (2007) were the first to extend flux balance analysis to a multispecies context, using a multi-compartment flux balance model to predict metabolite exchange between the sulfate reducer *Desulfovibrio vulgaris* and the methanogen *Methanococcus maripaludis*, thereby highlighting the important role of hydrogen in syntrophy between the two microbes. Since this pioneering study, metabolic models have been used to investigate potential interactions within several different microbial community contexts, with a few recent key studies highlighted in Table 1.

Plants present a unique challenge for the construction of high-quality metabolic models due to large eukaryotic genomes, multiple differentiated tissues, and redundancy associated with polyploidy in many plant genomes. As more genomic data is collected, current efforts to improve databases, annotations, and primary and secondary pathways (e.g., PlantSEED; Seaver et al., 2018) provide automated platforms as a starting point for plant-specific modelling. To date, metabolic models have been developed for specialized tissue types of several different plant species, including soybean seed (Moreira et al., 2019), rapeseed (Pilalis et al., 2011), rice (Chatterjee and Kundu, 2015), potato (Botero et al., 2018), and maize (Saha et al., 2011). Integration of multiple compartments within a metabolic model has also allowed reconstruction of multi-tissue models for more accurate representation of plant metabolism (Shaw and Cheung, 2020); for example, one of the most comprehensive plant models developed thus far has encompassed six different key tissues for *Arabidopsis* to more accurately predict whole-plant physiological responses (Gomes de Oliveira Dal’Molin et al., 2015). Additionally, multi-scale models (e.g., incorporation of gene regulation or phenomic data) can improve accuracy and provide experimental validation, aiding in understanding physiological effects of factors such as genome redundancy and circadian rhythm (Jez et al., 2021; Krantz et al., 2021).

Despite these advances in modelling applications for both plants and microorganisms, we found no studies investigating plant-microbe interactions with a community metabolic model approach (i.e., integrating both plant and microbial models into the same simulation). Recent algal-bacterial community models are moving in this direction to study more complex phototroph-heterotroph and eukaryote-prokaryote interactions (Dittami et al., 2014; Gomez et al., 2021). Furthermore, there still exists a large gap between modelling, lab-scale experimental results, and field-scale results, offering abundant opportunity for advances in this area. The sections below outline four key areas in which modelling could be used to enhance plant microbiome studies harnessing the utility of synthetic communities, followed by overall challenges and opportunities moving forward.

### Disentangling Multi-Directional Microbe–Microbe Interactions

**Figure 1:**
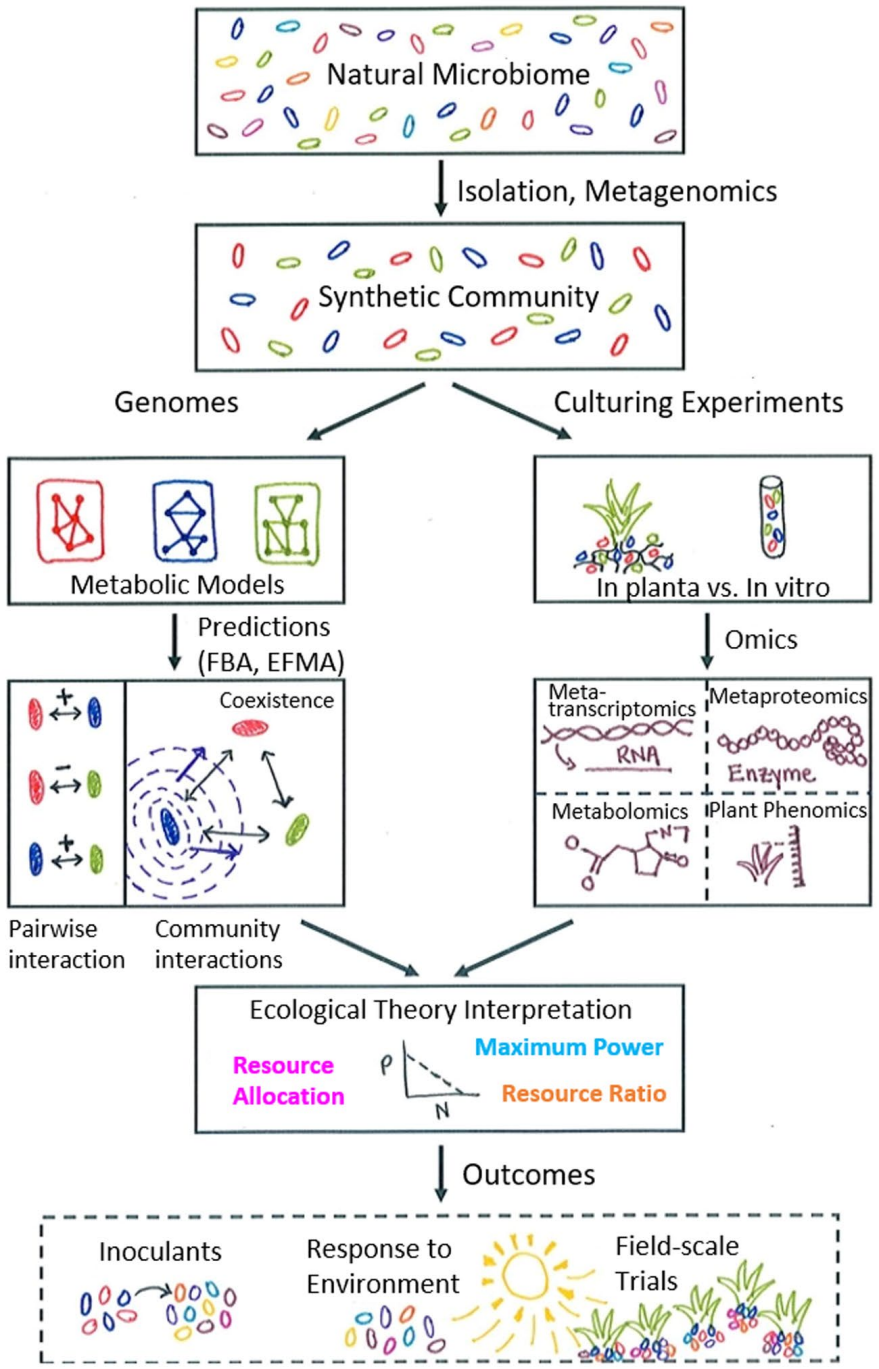
Schematic illustrating proposed metabolic modelling workflow with synthetic communities as input. The natural microbiome is a complex environment and is simplified to a synthetic community with a tractable number of microorganisms *via* experimental isolation techniques and/or metagenomics methods. Genomes for this limited number of microorganisms are used to construct metabolic models for each species, which can then be used with pathway simulation tools (flux balance analysis, FBA; elementary flux mode analysis, EFMA) to predict both pairwise and community interactions to observe changes due to additional community members and determine patterns of interaction. In the example shown, the blue microbe secretes a compound that is beneficial to both the red and green microbes and stabilizes their original negative pairwise interaction. Complementarily, culturing experiments can be used to examine the responses of microbial communities (both *in planta* and *in vitro*) to environmental variables such as limited nutrients or water stress. Collecting molecular data under specific experimental conditions provides data to integrate with modelling predictions, which can be interpreted with the aid of relevant ecological theory, such as resource ratio theory, resource allocation theory, or the maximum power principle. This process leads to iterative refinement of both models and experimental design, ultimately contributing to broader outcomes relevant to agricultural practice, such as the design of microbial inoculants to promote plant growth and resilience, an improved understanding of plant-microbe response to the environment under changing climate conditions, and the implementation of field-scale trials to further test interaction principles in the natural environment.

Interspecies interactions can take a variety of forms, e.g., mutualistic, commensal, antagonistic, parasitic (Holland and DeAngelis, 2009). Often, interactions are based on metabolite exchange which may be the basis for either an obligate or facultative relationship (Zelezniak et al., 2015; Beck et al., 2016). Even in simplified synthetic communities with few members, interactions are still complex and multifaceted. Interactions can sometimes be teased apart with carefully designed experiments; however, experimental measurements are not always able to distinguish the specific donor and recipient of metabolite exchanges. Modelling therefore allows for *in silico* testing of a multitude of possible unidirectional and bidirectional interactions within a community in a more rapid time frame. For example, a systematic approach for dissecting community interactions may involve first selecting specific pairs of organisms and predicting potential metabolite exchange-based interactions through a dual member model. Comparing the pairwise interaction predictions with simulations of a larger community will then aid in understanding how interactions could change with additional community member(s) (Figure 1).

### Modelling Plant-Specific Tissues and Community Assembly Processes

Rhizosphere and phyllosphere communities are complex assemblages of many species of organisms with the host plant, including not only bacteria but also archaea, fungi, and protozoa. The mechanism of community assembly is a major research question, including the effect of environmental stimuli on the assembly process, e.g., soil quality, nutrient availability, temperature, moisture, and presence of other organisms like pests and pathogens. Recruitment of microorganisms to a tissue involves metabolite exudation by the plant, but the ways in which plant-microbe and microbe-microbe interactions impact this process are currently poorly understood. Integration of plant and microbial metabolic models in a dynamic format (e.g., use of dynamic flux balance algorithms to investigate time-resolved interaction effects) will aid in better understanding and predicting the assembly process, particularly in understanding what drives colonization differences among plant species as well as how pathogens may disrupt the colonization process.

### Interaction Effects Resulting From Abiotic and Biotic Stresses

Beyond initial community assembly, environmental stresses are also known to have profound impacts on plant physiological responses. The literature is filled with examples demonstrating effects on growth and productivity by factors such as drought, temperature, and nutrient limitation (Cramer et al., 2011; Fahad et al., 2017). However, research has focused primarily on plant molecular and physiological responses, whereas many of these responses are mediated by plant-microbe and microbe-microbe interactions. Our understanding of how interactions change under different environmental stressors can benefit from modelling investigations, which can test a particular environment as part of the research question. For example, in flux balance analysis, substrate availability or uptake can be restricted according to the desired environmental scenario. Elementary flux mode analysis commonly examines pathway efficiency, and under nutrient limitations, cells are typically presumed to use the limited nutrient more efficiently relative to other substrates, guiding the simulation analysis. Careful design of simulation environments can be used in conjunction with greenhouse or laboratory experiments to gain a better understanding of how environmental pressures impact plant-microbe systems *via* community interaction effects. Biotic stressors can be investigated similarly (e.g., expanding a community model to include a pathogen).

### Incorporating Community Ecology Theory in Metabolic Modelling

Metabolic modelling uses genomic data combined with biochemical reactions governed by steady state conservation of mass to predict metabolite flux through a cell. However, this methodology further provides a platform for incorporating ecological theory in interpreting simulation results. For example, resource competition and allocation theories (Harcombe et al., 2014; Bernhardt et al., 2020) can be applied to predict how an organism might optimize its use of a certain resource, followed by comparison with efficient resource utilization predicted for competing species or the entire community. Similarly, resource ratio theory (de Mazancourt and Schwartz, 2010) examines tradeoffs between two key nutrients affecting growth, which can be expanded to examine resource usage within a community, as well (e.g., influence of microbial community composition on plant tissue allocation as observed in Henning et al., 2019). The maximum power principle, an alternative theory focused on energy usage, states that communities are evolutionarily organized to maximize energy consumption from the environment (Lotka, 1922; Sciubba, 2011). This principle has been less commonly applied in microbial communities but has been used to examine competition strategies (Cai et al., 2006; DeLong, 2008) and can provide another framework with which to evaluate the efficiency of community organization. Integrating these different community ecology theories can aid in elucidating the balance between competitive and cooperative interactions.

### Key Challenges in Plant Microbiome Modelling

Community models present a unique challenge as compared to individual species models when designing model simulations, specifically with regard to determining the optimization criterion that best represents the community objective in a natural system, as accurately modelling the overall community “goal” can be challenging. Typically for single species models, growth rate is used as the optimization criterion to determine a metabolic route that promotes maximum biomass formation, which may or may not be reflective of actual cellular targets, depending upon the environment. Many natural environments are nutrient-poor and may not promote growth at the highest capacity; desirable metabolic routes may instead optimize for efficient use of a limiting resource like nitrogen or for efficient energy production. Many current community modelling configurations optimize the overall community growth rate (Zomorrodi and Maranas, 2012). According to ecological principles, however, communities may not always be operating to achieve the maximum total biomass; thus, the objectives relevant to the specific scenario must be carefully decided upon when designing a model simulation. Objectives should be based on ecological principles and should account for the conditions or treatments being investigated.

Experimental validation of model predictions is essential due to the complexity of microbial communities and the underdetermined nature of individual metabolic networks which allows multiple possible solutions. With the broader availability of omics technologies and the ability to generate large data sets at lower costs, it is now possible to obtain multiple layers of data to complement modelling studies. Actualization of genomic potential can be examined at many levels – e.g., metatranscriptomics (Bashiardes et al., 2016), metaproteomics (Kleiner, 2019; Salvato et al., 2021), metabolomics, and plant phenomics. These different expression levels are important for understanding experimentally how the intersection of multiple metabolic networks affects the plant, as using one data type alone may yield very different predictions (Blazier and Papin, 2012; Payne, 2015). A key area of algorithm development is the incorporation of omics data within a metabolic network to influence optimization results to more accurately reflect and predict the outcome of a particular experimental scenario (e.g., Jungreuthmayer et al., 2013; Gerstl et al., 2015; Tian and Reed, 2018).

## Conclusion and Outlook

The above narrative addresses the current state of metabolic modelling as applied specifically to plant-microbe-environment interactions, detailing some of the key challenges in expanding modelling applications to these complex systems. Merging computational and experimental approaches will improve our limited understanding of multi-faceted plant-microbe-environment relationships (Figure 1). Advances will enable agricultural improvements, such as development of microbial inoculants to promote plant growth or resilience in the face of increasing global climate events. With these efforts, maintaining quality standards for modelling across the scientific community (Carey et al., 2020; Lieven et al., 2020) will continue to be essential when extending models and algorithms to more complex plant-microbe systems.

Envisioned future applications (Figure 1) involve starting from a relatively small synthetic microbial community (e.g., less than 10 members), developing a genome-enabled model for each member, validating individual models with experimental data (as much as possible), and investigating *in silico* pairwise interactions between species. Successively larger community models can be constructed by including additional member(s) and comparing with the previous pairwise models to observe how predictions change as more members are added. Laboratory and greenhouse experiments testing different community compositions, environmental factors, or other variables of interest can be used to verify and refine the community model, with the ultimate goal of moving to more complex systems (e.g., field-scale validation in agricultural settings). With continued effort and progress, the general principles and pattern of interactions uncovered through this systematic process is anticipated to be transferable to other plant systems; the microbes involved in the interactions may be different but likely follow similar governing interaction rules and can be used to engineer agricultural solutions in many different crops.

## Data Availability Statement

The original contributions presented in the study are included in the article/supplementary material; further inquiries can be directed to the corresponding author.

## Author Contributions

AB conceived the study, performed literature review, and prepared the initial draft of the manuscript. MK and A-KG performed literature review and curated the draft manuscript. All authors contributed to the article and approved the submitted version.

## Funding

This work is supported by Agriculture and Food Research Initiative Agricultural Microbiomes grant no. 2021-67013-34537 from the USDA National Institute of Food and Agriculture.

## Conflict of Interest

The authors declare that the research was conducted in the absence of any commercial or financial relationships that could be construed as a potential conflict of interest.

## Publisher’s Note

All claims expressed in this article are solely those of the authors and do not necessarily represent those of their affiliated organizations, or those of the publisher, the editors and the reviewers. Any product that may be evaluated in this article, or claim that may be made by its manufacturer, is not guaranteed or endorsed by the publisher.
